# Short stays, high needs: gender disparities in Czech forensic psychiatric inpatient care

**DOI:** 10.3389/fpsyt.2025.1604957

**Published:** 2025-10-23

**Authors:** Marek Páv, Jaroslav Pekara, Jáchym Valeš, Jiří Závora, Dominik Korený, Michaela Zahrádka-Köhlerová, Martin Anders, Eva Kitzlerová

**Affiliations:** ^1^ Department of Psychiatry, First Faculty of Medicine Charles University in Prague and General University Hospital in Prague, Prague, Czechia; ^2^ Department of Psychiatry, Bohnice Psychiatric Hospital, Prague, Czechia; ^3^ Medical College, Prague, Czechia; ^4^ Department of Psychology, Faculty of Arts, Charles University, Prague, Czechia; ^5^ Institute of Forensic Sciences, Prague, Czechia

**Keywords:** forensic psychiatry, women needs, HoNOS-secure, MOAS (Modified Overt Aggression Scale), inpatient forensic treatment, female forensic population, inpatient violence, gender differences

## Abstract

**Introduction:**

Limited research has explored the sociodemographic profiles, institutional behaviours, and treatment needs of women receiving forensic psychiatric care, particularly in Eastern Europe. Existing evidence suggests that women differ from men in several clinically significant ways that impact service delivery, treatment strategies, and overall care.

**Methods:**

A cross-sectional design was employed involving 85 women and 753 men across 14 forensic psychiatric facilities in the Czech Republic. Data were collected at two-time points, six months apart, using the Health of the Nation Outcome Scale-Secure (HoNOS-secure) and the Modified Overt Aggression Scale (MOAS). Gender differences in psychiatric diagnoses, length of stay (LoS), aggression, and unmet needs were statistically analysed.

**Results:**

The mean age of women was 45.08 years, compared to 41.43 years for men. Women had a significantly shorter average LoS (893.27 days; SD 116–3935) than men (1358.36 days; SD 28–15311). Women were more often diagnosed with psychotic and substance use disorders, had higher rates of violent index offences, and were 2.33 times more likely to receive antipsychotic medications. Although women initially demonstrated higher MOAS scores, they showed significant improvement over time. Both genders exhibited reduced security needs at follow-up. Nonetheless, high levels of unmet needs remained, particularly among women.

**Conclusions:**

The findings emphasise the importance of gender-responsive approaches in forensic psychiatric care. Establishing specialised forensic units for women is crucial to addressing their distinct clinical and psychosocial needs, enhancing treatment outcomes, and reducing recidivism. This study identifies critical service delivery gaps and reinforces the need to develop targeted interventions tailored to women in forensic settings.

## 1 Introduction

Women remain consistently underrepresented in prisons and forensic psychiatric settings compared to men, although their proportions vary significantly across countries. For instance, women account for only 5–6% of the forensic population in Germany (1), while in Canada, this figure rises to approximately 15% (1–3). This demographic disparity likely results from a complex interplay of factors, including variations in criminal history, individual and contextual characteristics, access to healthcare services, correctional system structures, and legal frameworks across jurisdictions (4, 5). These differences may also reflect fundamental distinctions between male and female forensic populations, which carry important clinical implications. Although women make up a small fraction of individuals in global prison and forensic psychiatric populations, recent decades have seen a notable rise in violent offences committed by women and increased admissions to forensic psychiatric care (6, 7).

Length of forensic stay is a critical metric in forensic psychiatry, balancing the need for comprehensive treatment of individuals with mental illness and the ethical obligation to limit deprivation of liberty to what is strictly necessary (4, 8). Similar to gender-based disparities in placement rates, differences in the length of stay (LoS) for women in forensic institutions also vary. For example, the average LoS is approximately 19 months in the UK (9) and Switzerland (10), but can be significantly longer in other contexts. Several international studies examining gender-based differences in forensic psychiatric LoS have yielded consistent results. A long-term Swedish study reported a median LoS of 80.7 months for women and 94.1 months for men (11). Similarly, a Dutch multicentre study recorded average durations of 54.9 months for women and 55.7 months for men (12). Irish research also aligned with these findings, showing a median forensic LoS of 80.7 months for women and 94.1 months for men (13). Notably, while these studies consistently show a trend towards slightly longer stays for men compared to women in forensic psychiatric care, none of the observed differences reached statistical significance. This pattern suggests that gender may have a minimal impact on LoS in forensic psychiatric settings.

The applicability of male-centred research findings to female forensic populations remains questionable, particularly given the limitations of existing small-sample studies (1, 7, 14–17). Women are increasingly conceptualised as a distinct subpopulation within forensic mental health services, with unique sociodemographic and clinical characteristics (18). However, current research often overlooks factors specific to female criminality (17). Some evidence suggests that women are more likely to have substance use disorders (7, 16)), receive a diagnosis of borderline personality disorder (16, 19), and report extensive histories of psychiatric treatment compared to men (20). Occupational and behavioural differences are also evident; women are less likely to be in full-time employment, more likely to work in casual roles (21), and tend to display distinct criminal behaviour patterns (17).

Additional findings highlight the extent of psychosocial vulnerability among women in forensic settings. Two-thirds are not engaged in a stable relationship, over half lack a high school diploma, and three-quarters were unemployed prior to incarceration (22). Another study found that 39% of women were homeless before hospitalisation (19). While individuals with severe mental illness across genders commonly experience victimisation (23), women are more likely to have suffered sexual abuse (24). This victimisation is associated with a wide range of mental health issues—including substance abuse, depression, and anxiety (25)—as well as socioeconomic challenges such as poverty, homelessness, and limited educational and employment opportunities (7, 26). Addressing the trauma histories of justice-involved women is, therefore, essential. To meet thiese needs, programs such as Beyond Trauma and Beyond Violence have been established (27). In many cases, trauma manifests as internalising behaviours—such as depression, PTSD symptoms, and substance use disorders— as well as maladaptive or violent behaviour (25). The consequences often extend intergenerationally: 54% of women admitted to forensic facilities have children, and in 81% of those cases, their children were removed by social services prior to the women’s admission (12).

Gender differences are also evident in patterns of criminal behaviour. Drug-related offences are more prevalent among female forensic patients than among their male counterparts and are closely associated with substance use disorders (19). Contrary to traditional assumptions about gendered violence, women in forensic settings are more likely than men to be convicted of serious violent offences such as murder or arson (13, 21). Some studies even suggest that female patients are just as likely as males to commit violent index offences or exhibit inpatient aggression (7, 26). However, the nature of violence among women differs considerably from that of men; it tends to be indirect, reactive, and relationally driven rather than instrumental or premeditated (7, 16). Moreover, violence in this context is often underpinned by psychotic phenomena and is frequently directed against relatives (28). Despite these distinctions, other studies report similarities between male and female forensic populations in variables such as age at incarceration, type of employment, and psychiatric treatment history (29, 30).

It is well established that forensic psychiatric patients experience higher levels of unmet needs compared to those in general psychiatric (31). Standardised instruments such as the Camberwell Assessment of Need–Forensic Version (CANFOR) (32) and the Health of the Nation Outcome Scale-Secure Version (HoNOS-secure) (33) have been developed to accurately assess these needs. The HoNOS-secure, which integrates general psychiatric evaluation with a seven-item subscale for secure environments, demonstrates strong interrater reliability and is widely used in routine clinical practice (34, 35). Nevertheless, assessment results often vary depending on the evaluator’s perspective: staff tend to report a greater number of total needs, while patients are more likely to identify unmet needs—underscoring the importance of greater patient involvement in planning service provision (15, 36). Although research has contributed to our understanding of needs in forensic populations more broadly, few studies have examined the specific health and social needs of women in forensic settings. As a result, these needs risk being overlooked or inadequately addressed (1, 29). This research gap not only raises questions about the generalisability of male-centred findings but also highlights the need for targeted studies aimed at developing gender-specific treatment frameworks.

In the Czech Republic, individuals with mental health conditions may be ordered to undergo forensic treatment if a court determines that their criminal responsibility was diminished or absent due to the influence of their mental state at the time of the index offence (36). Forensic treatment is mandated based on expert assessment and may take the form of inpatient or outpatient (community-based) care. While a formal risk assessment is not required to specify protective measures, the decision is guided by the clinical judgement expressed in expert opinions and the court’s consideration of the index offence and other contextual factors.

The Czech Republic’s forensic mental health system operates through a network of 14 general psychiatric hospitals, each responsible for a specific catchment area (36). This system follows a dual model of inpatient forensic care, comprising both specialised wards for specific patient groups—such as sex offender programs (37) —and mixed units housing both forensic and general psychiatric patients. A significant gender disparity exists within this framework: while men have access to specialised forensic units tailored to particular diagnoses, women undergoing court-ordered forensic treatment are typically placed in general psychiatric wards without specialised care options. The prison system supplements the healthcare network by offering limited forensic placements, including approximately 200 inpatient beds and 90 high-security posts within prison-based services (36). There is considerable regional variation in the number of patients per 100,000 residents, with significant inter-hospital variations in LoS associated with index offences, diagnoses, or treatment programs (38). In Czechia, longer forensic stays are associated with older age, longer psychiatric hospitalisation histories, higher doses of antipsychotic medication (expressed in olanzapine equivalents), clozapine prescriptions, psychosocial dysfunction, diagnoses of psychotic or paraphilic disorders, and sexual index offences (39). Conversely, shorter LoS are linked to factors such as stable interpersonal relationships, employment prior to hospitalisation, supportive personal networks, and index offences involving substance use (40). Previous findings identified several factors influencing discharge likelihood from forensic facilities. Decreases in HoNOS-secure scores correlate with a higher probability of discharge, indicating that improvements in mental health and risk management are key to release (41). Additionally, diagnoses of substance use disorders tend to increase the likelihood of discharge, while intellectual disability diagnosis significantly decreases discharge probability.

This cross-sectional, representative sample study aimed to compare the characteristics of female and male subgroups within the Czech Republic’s inpatient forensic population, with a focus on sociodemographic profiles, treatment-related variables, and gender-specific needs.

## 2 Material and methods

### 2.1 Participants and procedures

This study was conducted as part of the Ministry of Health’s deinstitutionalisation initiative in the Czech Republic and was integrated into the CENZUS cross-sectional survey. The survey aimed to collect comprehensive data from all inpatient forensic patients across the 14 psychiatric institutions in the country that provide inpatient forensic care. These facilities operate under the jurisdiction of the Ministry of Health and are categorised as its “directly managed” organisations. The inpatient forensic system in Czechia is discussed in detail below, with an analysis of inter-hospital diffences (38).

Participation was offered to all 877 inpatient forensic patients hospitalised by 1 July 2021; 36 patients refused to participate. Consequently, data were collected from a total of 841 patients. The final sample consisted of 85 women and 753 men; the remaining three patients were excluded because their gender could not be determined during the dataset cleaning process. The sample size for each analysis varies due to missing-at-random data. This missing data may have been caused by issues during the collection process at the participating hospitals, which were beyond our control. Although the design of our study is primarily cross-sectional, data collection was conducted at two separate time points to fit the MOAS use-case. At the first session, we collected all data except for the MOAS results, which we collected at a later date. Only HoNOS results were collected at both sessions to assess between-gender and within-gender differences at different time points. This allowed us to test for improving or worsening HoNOS scores over time.

An extensive checklist was developed based on a literature review and prior experience with studies conducted on the forensic population in Czechia (40, 42). Teams within the respective hospitals conducted the coding process as part of the deinstitutionalisation project overseen by the Ministry of Health. The evaluators were professionals in the mental health field, typically qualified in nursing, social work, or working as junior psychologists. All coders received training on dataset handling and item coding, and additional clarification sessions were held throughout the project to ensure consistency and accuracy in data collection. Trained evaluators assessed HoNOS-secure scores. Data were collected from multiple sources, including electronic medical records, formal assessments, physical files, and interviews with patients and direct-care staff. Sociodemographic variables (relationship status, employment status, and education level) and clinical characteristics (diagnosis, LoS, and index offence) were obtained from medical records, while information on index offences and criminal history was extracted from official court documents. Clinical observations were assessed by a trained evaluator who reviewed medical records and conducted interviews with direct-care staff. All diagnoses were coded according to ICD-10 (International Classification of Diseases–10^th^ revision) (43). The department team and the head physician of each respective department were responsible for confirming the primary diagnosis.

### 2.2 Measures

#### 2.2.1 Health of the Nation Outcome Scale for Users of Secure and Forensic Services

HoNOS-secure is a specialised risk assessment tool used in secure and forensic mental health services for both men and women (44). It was developed through collaboration between the Royal College of Psychiatrists and the UK Department of Health (45). It extends the original HoNOS by including a seven-item safety scale (46) that assesses the current need for secure or forensic care, focussing on risks of harm to self or others. HoNOS-secure offers a comprehensive view of a client’s clinical presentation and security needs, supporting decisions on placement, treatment, and risk management (47). Each item is scored from 0 (no problem) to 4 (severe problem), with a score of 1 or more on the secure subscale indicating a need for advanced risk management. The HoNOS also measures service effectiveness in four domains: behavioural problems, cognitive and physical impairment, symptomatic problems, and social difficulties—in both men and women. The HoNOS-secure was adapted for the Czech Republic in a 2009 pilot study (48). The HoNOS used in this study was adapted for the Czech Republic in a 2009 pilot study (48). That study showed statistically significant correlations with the subscales of the Czech-adapted GAF Global Assessment of Functioning), with r values between -0.496 and -0.72. The validation methods used in the pilot study were similar to those used by the authors of the original HoNOS (48).

#### 2.2.2 Modified Overt Aggression Scale

Clinicians use tools such as MOAS to assess patients’ aggressive behaviours in both men and women (49, 50). The MOAS measures the frequency and severity of aggression on a scale of 0 to 40, with higher scores indicating greater aggression. They are often used in research settings to monitor changes over time. We used the instrument to retrospectively record violent incidents of all kinds over three months of data collection.

### 2.3 Data analysis

Data were processed using MS Excel 2016, and statistical analyses were conducted using R version 4.0.5 (R Project for Statistical Computing, Vienna, Austria). We used Jamovi (version 2.3.28) to generate descriptive and frequency tables. Student’s t-test, Mann–Whitney U test, Pearson’s chi-squared test, Fisher’s exact test, and Friedman’s test were employed to compare characteristics between male and female groups. To address issues related to multiple comparisons, significance levels for each test group (i.e., sociodemographic, clinical, and legal characteristics) were adjusted using the Benjamini and Hochberg procedure (51). A p-value ≤ 0.05 was considered statistically significant.

During statistical processing, some variable categories were merged to reduce data loss due to low response frequencies. For example, the Czech education system differentiates among several types of upper secondary education based on specialisation and final examination. These were grouped under a single category, “completed upper secondary education.” Descriptive tables were generated to report calculated means, standard deviations (SDs), relative frequencies, and N counts across different time points. Separate tables were created for men and women to examine gender differences. Age (years), length of stay (days), duration of primary diagnosis (years), HoNOS subscale scores, and MOAS scores were treated as quantitative variables. Gender, criminal liability, different types of medication or no medication, ward security level, relationship status, employment status, highest education attained, contact with relatives, housing options, pathways before inpatient forensic treatment, and diagnoses based on the ICD-10 were treated as qualitative variables. For Fisher’s exact 2×2 test, medication and diagnoses were dichotomised into individual binary variables, e.g. ‘treated with antipsychotics’ (yes/no) or ‘diagnosed with F20-29’ (yes/no).

### 2.4 Ethics

The study received approval from the Ethics Committee of Bohnice Psychiatric Hospital and adhered to both national legislation and institutional guidelines. All participants provided written informed consent prior to enrolment. Collected data were anonymised, with individuals identified solely by treatment numbers and hospital identifiers. These identifiers remained confidential during all external data processing.

## 3 Results

The sociodemographic characteristics of the participants are summarised in **Table 1**. The mean age was 45.08 years in the female subgroup and 41.43 years in the male subgroup, with a significant gender difference (p = .018). Length of stay also differed significantly between groups (p = .003), with a mean of 893.27 days for women and 1358.36 days for men. Approximately half of both women and men were diagnosed with psychotic disorders (ICD-10 F2): 47 women (55.29%) and 344 men (45.68%), a statistically significant difference (p = .039). A substantial proportion of participants—21.18% of women and 17.66% of men—were also diagnosed with a substance use disorder (ICD-10), although this difference was not significant. Among men, 23.77% had a primary diagnosis of a personality disorder or paraphilia (ICD-10); due to the small number of women (n = 3) in this category, statistical comparison was not feasible. No significant gender difference was observed regarding illness duration. The sample overall reflects a low socioeconomic status, with 78.87% of women and 82.54% of men either unemployed or receiving disability allowances before entering forensic treatment. Nonetheless, women demonstrated significantly higher educational attainment (p <.001), being 4.24 times more likely to have a university degree. Women also showed slightly better housing conditions and more frequent social contact, though these differences were not statistically significant. Additionally, women were significantly more likely to be retired and receive a pension (p = .026). No gender difference was found in terms of legal incapacity.

**Table 1:** General characteristics of female and male forensic psychiatric patients.

Age (years)	Women (n=85)	Men (n=753)	P-value
Mean SD	**45.08**	**41.43**	**0.018^M^**
Range	19 - 79	16 - 85	
Length of Stay (days )	Women (n=85)	Men (n=753)	P-value
Mean SD	**893.27**	**1358.36**	**0.003^M^**
Range	116 - 3935	28 - 15311	
Main Diagnosis Duration (years)	Women (n=83)	Men (n=731)	P-value
Mean (SD)	12.506 (11.447)	13.005 (12.090)	.795
Diagnoses due ICD-10, n (%)	Women (n=85)	Men (n=750)	P-value
F00-F09 Physiological Disorders	6 (7.06)	32 (4.25)	Ns
F10-F19 Disorders due to Substance use	18 (21.18)	133 (17.66)	Ns
F20-F29 Psychotic disorders	**49 (55.29)**	**344 (45.68)**	**.039^A^**
F30-F39 Mood [affective] disorders	1 (1.18)	4 (0.53)	NA
F40-F49 Stress-related Disorders	1 (1.18)	1 (0.13)	NA
F50-F59 Behavioural syndromes	0 (0.00)	2 (0.27)	NA
F60-F69 Personality Disorders and Paraphilia	3 (3.53)	179 (23.77)	NA
F70-F79 Intellectual disabilities	7 (8.23)	53 (7.04)	Ns^A^
F80-F89 Developmental Disorders	0 (0.00)	2 (0.27)	NA
Relationship status, n (%)	Women (n=77)	Men (n=735)	P-value
Without stable relationship	70 (90.91)	703 (95.65)	Ns
Married, in a relationship	7 (9.09)	32 (4.35)	Ns
Employment Status, n (%)	Women (n=85)	Men (n=739)	P-value
Disability pension	40 (47.05)	358 (48.44)	Ns
Unemployed	33 (38.82)	252 (34.10)	Ns
Voluntary, part-time job, own accounted worker	2 (2.35)	47 (6.36)	NA
Stable job profile (at least a half-time job)	2 (2.35)	52 (7.04)	NA
Retirement pension	**8 (9.41)**	**30 (4.06)**	**.026^A^**
Highest Obtained Education, n (%)	Women (n=85)	Men (n=752)	P-value
Elementary school (6–15 years), (9 years)	38 (44.71)	393 (52.26)	Ns
Upper secondary education (15–19 years), (4 years)	39 (45.88)	341 (45.35)	Ns
College degree (19 +), (3–8 years)	**8 (9.41)**	**18 (2.39)**	**<.001^A^**
Contact with relatives, n (%)	Women (n=85)	Men (n=753)	P-value
Has contact with someone	70 (82.35)	581 (77.16)	Ns
No contact	15 (17.65)	172 (22.84)	Ns
Housing Options n (%)	Women (n=79)	Men (n=715)	P-value
Homeless or with a temporary living place	26 (32.91)	254 (35.52)	Ns
Social facilities	16 (20.25)	151 (21.12)	Ns
Own flat, living with relatives	37 (46.84)	310 (43.36)	Ns
Legal responsibility	Women (n=85)	Men (n=753)	P-value
Full	42	454	Ns
Diminished	36	268	Ns
Re-evaluation in progress	7	31	Ns
Pathway before FT, n (%)	Women (n=85)	Men (n=750)	P-value
Community Forensic Treatment	15 (17.65)	133 (17.73)	NsA
Another psychiatric ward	8 (9.41)	93 (12.40)	NsA
Other	2 (2.35)	29 (3.87)	NA
Community (without forensic treatment)	**48 (56.47)**	**276 (36.8)**	**<.001^A^**
Imprisonment	**10 (11.76)**	**208 (27.73)**	**.002^A^**
Secure detention	2 (2.35)	14 (1.87)	NA

Both parametric and nonparametric methods were used to achieve a better fit for the data. M, Mann-Whitney U test; A-p-values by A Chi-square test. Ns, not statistically significant. Missing data n=3. The sample size varies across methods: for females, N ranges from 77 to 85; for males, N ranges from 715 to 753. The bolded numbers are statistically significant.

For additional sociodemographic details, refer to **Table 1**. There was a significant gender difference in the context of admission: women were more likely to have lived in the community prior to forensic hospitalisation (p <.001), while men were more likely to have been transferred from prison (p = .002). Women had 2.24 times greater odds of being hospitalised from the community, whereas men were 2.86 times more likely to be transferred from prison.

No significant gender-related differences were identified in terms of criminal liability. However, analysis of index offences revealed that women were 1.62 times more likely than men to receive forensic treatment for violent offences (p = .041; see **Table 2**). As expected, 21.09% of male participants had been charged with sex offences, while no such charges were recorded for women. During treatment, women were 2.38 times more likely to be placed in a low-security environment, whereas men were more likely to be housed in high-security units (p <.001). No significant gender difference was found in the use or absence of restraints. A significant difference (p = .005) was observed in the use of antipsychotic medication, with 84.71% of women receiving such treatment compared to 70.67% of men. This suggests that women in forensic inpatient care were approximately 2.33 times more likely to receive antipsychotic medication than men.

**Table 2:** Criminal characteristics and clinical variables of female and male forensic psychiatric patients.

Criminal liability, n (%)	Women (n=85)	Men (n=749)	P-value
Completely diminished	41 (48.24)	300 (40.05)	Ns
Substantially diminished	25 (29.41)	311 (41.52)	Ns
Not substantially diminished	19 (23.17)	138 (18.42)	Ns
Index Offence Category, n (%)	Women (n=85)	Men (n=749)	P-value
Violence	**33 (38.82)**	**211 (28.17)**	**.04^C^**
Homicide or homicide attempt	6 (7.06)	76 (10.15)	Ns
Sexual violence	0 (0.00)	158 (21.09)	NA
Property crime	12 (14.12)	82 (10.95)	Ns
Arson	3 (3.53)	8 (1.07)	NA
General criminality	31 (36.47)	214 (28.57)	Ns
Ward Security Level, n (%)	Women (n=85)	Men (n = 753)	P-value
High Security	**27 (31.76)**	**396 (49.9)**	**<.001^C^**
Low Security	**52 (61.18)**	**320 (51.1)**	
Absconsion, n (%)	Women (n=77)	Men (n=701)	P-value
Yes	5 (6.49)	34 (4.85)	Ns
No	72 (93.51)	667 (94.14)	
Was under Restriction, n (%)	Women (n=79)	Men (n=716)	P-value
Yes	2 (2.53)	30 (4.19)	NA
No	77 (97.47)	686 (95.81)	
Medication within the Treatment, n (%)	Women (n=85)	Men (n=750)	P-value
Antipsychotics	**72 (84.71)**	**530 (70.67)**	**.005^C^**
Antidepressants	5 (5.88)	27 (3.60)	Ns
Antiandrogens	0 (0.00)	142 (18.93)	NA
Mood stabilisers	0 (0.00)	14 (1.87)	NA
No medication	8 (9.41)	40 (5.33)	Ns

C, Chi-square test. Ns, not statistically significant. The sample size varies across methods and demographics: for females, N ranges from 77 to 85; for males, N ranges from 701 to 753. The bolded numbers are statistically significant.

The next stage of analysis examined gender differences in inpatient violence, as recorded by the MOAS, refer to **Table 3**. Among 716 men and 79 women, a significant reduction in violence was observed in the female subgroup (p = .014), with average scores dropping from 1.97 to 1.11 points. Further analysis revealed a significant difference (p = .025) at the first data collection, with women scoring an average of 2.07 points compared to 1.69 points in men, though no difference was found at the second collection point. Analysis of treatment needs, assessed using the HoNOS scale (see **Table 4**), showed a mean total score of 11.61 at the first data collection and 11.75 at the second for the full sample. Although the behavioural, symptomatic, and social subscales revealed significantly higher (i.e., worse) scores over time, the overall change in the total score was not significant. This trend was mirrored in the male subgroup, while no significant change was noted in the female subgroup.

**Table 3:** Assessment of violent behaviour using the modified overt aggression scale in male and female forensic psychiatric patients.

Whole sample (n=798)	First data collection	Second data collection	P-value
Mean (Sd)	1.68 (3.83)	1.53 (3.84)	Ns
Range	0-28	0-26	
Women (n=79)			
Mean (Sd)	**1.97 (4.10)**	**1.11 (3.15)**	**.014^W^**
Range	**0-20**	**0-18**	
Men (n=716)			
Mean (Sd)	1.65 (3.80)	1.58 (3.91)	Ns
Range	0-28	0-26	
First data collection (n=838)	Women (n=85)	Men (n=753)	P-value
Mean (Sd)	**2.07 (4.15)**	**1.69 (3.95)**	**.025^M^**
Range	**0-20**	**0-28**	
Second data collection (n=795)	Women (n=79)	Men (n=753)	
Mean (Sd)	1.11 (3.15)	1.58 (3.91)	Ns
Range	0-18	0-26	

M, Mann-Whitney U test; W, Wilcoxon rank test. Ns, not statistically significant. The sample size varies across genders and different time points: for females, N ranges from 92 to 85; for males, N ranges from 716 to 753. *The whole sample includes three patients with unknown genders. The bolded numbers are statistically significant

**Table 4:** Assessment of needs in female and male forensic psychiatric patients using the Health of the Nation Outcome Scales (HoNOS).

Whole sample (women + men)	First data collection mean	Second data collection mean	P-value
Total score (n=330)	11.61	11.75	Ns
HoNOS, behaviour subscale (n=564)	**1.42**	**1.77**	**<.001^RM^**
HoNOS, impairment subscale (n=368)	1.95	1.91	Ns
HoNOS, symptom subscale (n=548)	**2.94**	**3.21**	**.003^RM^**
HoNOS, social subscale (n=536)	**6.84**	**7.11**	**<.001^RM^**
Women			
Total score (n=31)	14.86	14.00	Ns
HoNOS behaviour subscale (n=60)	1.51	1.86	Ns
HoNOS, impairment subscale (n=38)	2.47	2.25	Ns
HoNOS symptom subscale (n=57)	3.40	3.50	Ns
HoNOS social subscale (n=54)	7.20	7.31	Ns
Men			
Total score (n=299)	11.36	11.60	Ns
HoNOS behaviour subscale (n=504)	**1.42**	**1.77**	**<.001^RM^**
HoNOS, impairment subscale (n=330)	1.90	1.87	Ns
HoNOS symptom subscale (n=491)	**2.89**	**3.19**	**.003^RM^**
HoNOS, social subscale (n=482)	**6.80**	**7.09**	**.002 ^RM^**

RM, Non-parametric Repeated Measures ANOVA was used. Ns: not significant. The sample size varies across different time points and subscales: for females, N ranges from 31 to 60; for males, N ranges from 299 to 504. The bolded numbers are statistically significant.

As shown in **Table 5**, scores on the “secure” subscale of the HoNOS-secure also improved significantly (p <.001) in the full sample and the male subgroup by the second data collection. However, no significant improvement was observed in the female subgroup. While men had significantly worse scores (p = .008) at the first collection point—averaging 7.17 compared to 6.04 in women—no significant gender difference was observed at the second time point.

**Table 5:** Assessment of security needs in female and male forensic psychiatric patients using the 'secure' subscale of the Health of the Nation Outcome Scales (HoNOS-secure).

Whole sample (n=688*)	First data collection	Second data collection	P-value
Mean (Sd)	**7.34 (3.13)**	**6.89 (3.09)**	**<.001^W^**
Range	**0-19**	**0-17**	
Women (n=72)			
Mean (Sd)	6.53 (3.62)	6.17 (3.41)	Ns
Range	0-14	0-14	
Men (n=613)			
Mean (Sd)	**7.45 (3.06)**	**6.99 (3.04)**	**<.001^W^**
Range	**0-19**	**0-17**	
First data collection (n=830)	Women (n=85)	Men (n=745)	P-value
Mean (Sd)	**6.04 (3.68)**	**7.17 (3.25)**	**.008^M^**
Range	**0-14**	**0-19**	
Second data collection (n=693)	(n=72)	(n=621)	
Mean (Sd)	6.17 (3.41)	6.96 (3.04)	Ns
Range	0-14	0-17	

M, Mann-Whitney U test; W, Wilcoxon rank test. Ns, not statistically significant. The sample size varies across genders and different time points: for females, N ranges from 72 to 85; for males, N ranges from 613 to 745. *The whole sample includes three patients with unestablished genders. The bolded numbers are statistically significant.

It is important to note that sample sizes and mean scores varied depending on whether patients participated in one or both data collections. Thirteen women were no longer present in the forensic psychiatric unit at the time of the second data collection, or their data were not captured. The full sample of 85 women had a lower mean score (6.04) compared to the subgroup from which these 13 individuals were excluded (mean = 6.53). This suggests that the excluded women may have exhibited less severe symptoms and/or experienced faster recovery, potentially leading to earlier discharge. A similar trend was observed in the male subgroup. However, due to the absence of follow-up data after discharge, any further interpretation remains speculative.

## 4 Discussion

The findings of this study offer valuable insights into gender-specific characteristics and needs among forensic psychiatric patients in the Czech Republic. Compared to men, women in forensic settings demonstrated distinct profiles, including shorter lengths of stay, a higher prevalence of psychotic and substance use disorders, and a greater likelihood of being mandated to forensic treatment for violent index offences. Women were also more frequently placed in lower-security wards and more often prescribed antipsychotic medications, reflecting notable gender-based differences in institutional treatment approaches. Despite these observed patterns, high levels of unmet needs persisted across the population—particularly among women—suggesting that current therapeutic strategies may inadequately address their complex requirements. These findings highlight potential systemic gaps in service provision that warrant targeted policy and clinical attention.

The mean age of participants was 45.08 years for women and 41.43 years for men. Notably, men were older at the time of the index offence—a trend also reported in a Canadian sample (2). Comparison with other forensic samples, however, shows a higher mean age in our sample; in Germany, for example, the mean age of women was 35 (10.22) years (17); in Sweden, 41 years (20). In India, the mean age of patients was 31.3 ± 7.9 years (52) and in Switzerland, the mean age of women was 41 years (19).

The overall length of stay (LoS) in our sample positions the Czech system as one with a medium-to-long LoS, similar to countries like Poland (4.48) (3). However, the LoS observed in the female subgroup was remarkably short. Previous research suggests that LoS in forensic inpatient care is influenced by several sociodemographic and clinical factors, including the type of index offence, presence of a severe violent offence, or a schizophrenia diagnosis—factors typically associated with prolonged hospitalisation (8, 11). Interestingly, our data diverge from prior findings that report no statistically significant gender-related differences in LoS (11–13). In our sample, women were more frequently mandated to treatment for violent offences and had a higher prevalence of schizophrenia, yet they experienced significantly shorter LoS than men. This observed discrepancy raises important questions about whether the principles of risk–need–responsivity (RNR) are being appropriately applied to female forensic patients in the Czech Republic. It may also have implications for risk management in community settings following discharge, particularly in light of recent data from Germany showing relatively high recidivism rates among women (53). Moreover, service organisation and provision likely contribute to these gender differences in LoS. Unlike many men, women ordered to forensic treatment are often placed inconsistently across general psychiatric wards, long-term care units, or substance abuse programs—reflecting the absence of dedicated forensic facilities for women. This may lead to early discharge, as existing programs often lack gender-responsive therapies, such as adapted versions of dialectical behaviour therapy or schema-focussed therapy (16). These limitations in treatment provision compromise both the therapeutic and security aspects of care for women. The lack of specialised programs for female forensic patients and their problematic placement within general psychiatric settings was also highlighted in a 2024 report by the Czech Ombudsman (54). Another factor potentially contributing to the observed gender disparities in LoS could be institutional perceptions of risk and behaviour. Prior research has shown that gender stereotypes significantly shape women’s experiences in correctional environments, influencing how their behaviour, education, and social roles are interpreted (55).

More than half of the women in our sample were diagnosed with psychotic disorders, followed by substance use disorders. In comparison, data from Italy indicate that psychotic disorders account for 40% of diagnoses, followed by personality and depressive disorders (14). Similarly, in the Netherlands (12) and Switzerland (19) psychotic and substance use disorders were also the most prevalent. Our sample is characterised by a predominance of psychotic and substance use disorders, with relatively fewer diagnoses of mood and personality disorders. Consistent with the diagnostic profile, antipsychotic medication use was high among women in our sample. Women were 2.33 times more likely to be prescribed antipsychotics than men. However, given that 84.71% of the overall sample received antipsychotic medication, it is possible that these drugs were also prescribed to individuals with other diagnoses, such as intellectual disabilities or substance use disorders. This raises important concerns regarding potential antipsychotic polypharmacy and its associated side effects (56, 57).

The duration of illness of our entire sample (women 12.506 years, men 13.005 years) is long and appears to be longer than that observed by Buongiorno (9.0 years for women and 11.8 years for men) (14) and Valença (28). Previous studies have shown that women in prison and forensic populations often have a higher rate of contact with mental health services due to pre-existing psychiatric diagnoses (8, 16, 58). As Hodgins (18) noted, women admitted to general psychiatric services often receive little to no assessment or intervention for antisocial or aggressive behaviour, which may contribute to their eventual progression into the forensic system. Our findings from the Czech population, particularly regarding long-standing mental health service contact and illness duration, support this observation. This highlights a potential systemic issue: the limited ability of mental health services to identify and intervene early with women who are at elevated risk for forensic involvement. Women with antisocial behaviour profiles also play a critical role in perpetuating intergenerational cycles of antisocial tendencies, as they are often mothers responsible for their children (12). Breaking this cycle requires a systemic approach, such as implementing programs aimed at reducing teenage pregnancies or strengthening parenting skills. However, no such targeted programs currently exist within the forensic care system in Czechia.

Analysis of relationship status revealed that most patients, both male and female, were not in a relationship. Employment and housing data further reflect the poor socioeconomic conditions of the forensic inpatient population. Nearly half of all patients received disability pensions, with minor gender differences: 33.47% of men and 38.82% of women were unemployed. These figures are notably lower than those reported by D’Orta (10), who found an employment rate of approximately 60% among incarcerated women. Interestingly, 82.35% of women in our sample maintained significant social support, challenging typical assumptions about social isolation in forensic populations. This is encouraging, given that social support is a well-documented protective factor against violent or criminal behaviour (59, 60). Most patients lived either in their own homes or with relatives (approximately 54–58% for both sexes), and around one-fifth had access to social services after release. Our findings also challenge some persistent misconceptions. For example, women in our sample exhibited higher levels of education than commonly assumed. This is consistent with data from Switzerland (10), which similarly contradicts stereotypes that incarcerated women are largely uneducated or lack vocational skills. Pathway analysis showed that most women resided in the community prior to forensic treatment and had not been subject to prior court-ordered community treatment. This suggests that, for many women, forensic intervention occurred without previous justice system involvement. In contrast, men were significantly more likely to have been imprisoned before being transferred to forensic treatment. One explanation is that many male participants were sex offenders with only partially diminished criminal responsibility and were, therefore, sentenced to prison before referral to forensic psychiatric care.

Analysis of institutional behaviour revealed minimal gender differences in our sample. Compared to other forensic cohorts, our findings suggest a relatively low application of restraints and few incidents of escape from treatment (7, 9, 15, 16). Previous research has indicated that women may exhibit violent behaviour at rates equal to or even higher than men, both during index offences and while in treatment (7), which aligns with our findings.

Specifically, we observed a higher prevalence of violence in index offences among women, as well as increased inpatient aggression compared to their male counterparts. Despite these trends, both subgroups showed statistically significant reductions in violent incidents between the two measurement points. However, differing perceptions of how women express violence may lead clinicians to underestimate the associated risks, as suggested by the occurrence of violent incidents during treatment. The use of gender-sensitive tools—such as the Female Additional Manual, which complements the structured HCR-20 assessment—may support more accurate, gender-informed risk assessment and management (61).

We observed that women were often mandated to inpatient forensic treatment due to violent acts of varying severity, often directed towards relatives (16, 28). The prevalence of violent index offences among female forensic patients in our sample contrasts with Streb’s findings, which reported lower levels of violence and a predominance of drug-related offences among women (17). The minimal occurrence of sexual violence as an index offence among women in our study aligns with previous research (7, 19, 26)). Judicial assessments indicated that over three-quarters of women had significantly impaired or absent criminal responsibility at the time of their index offences. This finding corresponds with the high prevalence of major psychiatric disorders and the resulting verdicts of *not guilty by reason of insanity* within the female subgroup.

Assessments using the 12-item HoNOS scale showed no improvement in needs fulfilment during treatment. The total score for unmet needs remained stable, while subscale scores increased insignificantly overall, with significant worsening in behavioural, symptomatic, and social domains. The unbalanced subgroup sizes likely explain the lack of progress observed in women and the minimal total score change in men. The overall level of unmet needs in the sample corresponded to admission-level needs reported by previous studies (42), and, compared with other cohorts, our population demonstrated a high degree of unmet treatment needs (34, 42). Additionally, data from a Czech hospital involving long-term hospitalised forensic patients showed a HoNOS total score of 7.7 points (54). Overall, the inpatient forensic population showed a high level of unmet needs, particularly in the social and symptomatic subscales. Notably, women exhibited higher levels of unmet needs than men, exceeding the average for the total sample.

The HoNOS-secure scale captures “forensic needs,” such as the requirement for guarded ward leave or the risk of harm to oneself or others (28, 41). Our findings showed that women demonstrated a lower need for security precautions and were 2.38 times more likely to be placed in low-security wards. This observation warrants attention, particularly given the high prevalence of violent index offences and manifestations of inpatient aggression. Across the entire sample, security needs significantly decreased between the two data collection points, although this shift was likely driven primarily by changes in the male subgroup due to unbalanced sample sizes.

This study has some limitations that should be acknowledged. First, the notable imbalance between male and female samples presents a primary limitation, as it may have introduced bias into the analyses. While comparing male and female forensic populations yields useful insights into gender-specific differences, the absence of a non-forensic female control group limits our ability to identify characteristics unique to forensic patients. Another limitation lies in the multicentre study design, which involved data collection by professionals with varying levels of expertise. This variability may have contributed to researcher bias. The cross-sectional design is another constraint, capturing only a point-in-time assessment rather than long-term outcomes, such as those at discharge or during community reintegration. Consequently, the findings offer only a snapshot of treatment experiences rather than a comprehensive understanding of patient trajectories. Furthermore, although the study identified high levels of unmet needs across the forensic population, it did not explore how systemic factors or resource allocation might address these gaps. Finally, the study was not designed specifically to examine women’s experiences, which left several important gender-related variables—such as trauma history, physical health, number of children, or caregiving roles—unmeasured. These omissions limit the depth of our gender-based analyses.

These limitations suggest several directions for future research. Longitudinal studies with more frequent assessments over extended periods would allow for more nuanced tracking of treatment progression and long-term outcomes. Targeted investigations into the therapeutic handling of trauma and its impact on recovery are essential for improving care for female forensic patients. Likewise, exploring parenting competencies and family dynamics remains an under-researched but important area. Given that many women in forensic settings are mothers—and that their children are frequently placed in social care—future studies could help illuminate intergenerational behavioural patterns and guide the development of interventions aimed at breaking cycles of trauma and maladaptive functioning.

## 5 Conclusion

This study sheds light on gender-specific differences in the characteristics, behaviours, and needs of forensic psychiatric patients in Czechia. Women in forensic settings had shorter lengths of stay, higher rates of psychotic and substance use disorders, and were more likely than men to be prescribed antipsychotic medication. They were also more frequently ordered to treatment for violent index offences and more often placed in lower-security wards, reflecting distinct clinical and institutional profiles. Despite these differences, high levels of unmet needs persisted across the population, with particularly pronounced gaps in social and symptomatic support among women. Our study represents one of the first from the Central and Eastern European region to focus specifically on the female forensic population, thereby complementing existing findings from Western, Northern, and other global contexts.

These findings underscore the urgent need for gender-responsive approaches in forensic psychiatric care. For example, gender-specific risk assessments remain absent in current practice, yet their implementation could improve gender-informed risk management. The lack of dedicated forensic units for women in Czechia further restricts opportunities for tailored care. Establishing gender-sensitive therapeutic programs and specialised facilities could significantly enhance treatment effectiveness, reduce recidivism, and help interrupt intergenerational cycles of antisocial behaviour.

In summary, addressing the gender-specific needs of women in forensic psychiatric care is essential for ensuring equitable service provision and improving outcomes for this vulnerable population. Future research should focus on systemic reforms that support tailored interventions while deepening our understanding of women’s unique clinical and social challenges.

## Data Availability

The raw data supporting the conclusions of this article will be made available by the authors, without undue reservation.
