# Supplementary material for: Short stays, high needs: gender disparities in Czech forensic psychiatric inpatient care
Source: Front Psychiatry. 2025 Oct 23;16:1604957. doi: 10.3389/fpsyt.2025.1604957 (PMC12589535; doi:10.3389/fpsyt.2025.1604957)
Supplement: Supplementary file 1 [file Table1.docx]

| **Index Offence, n(%)** pathway | **Women (n=85)** | **Men (n=753)** |
| --- | --- | --- |
| Crimes against official decisions (obstruction  of official decision, negligent criminal activity) | 11 (12.94) | 48 (6.37) |
| Other violent behavior | 17 (20.00) | 154 (20.45) |
| Other violent behavior +  Property-related criminal activity | 0 (0.00) | 4 (0.53) |
| Other violent behavior + Rioting (other  than exhibitionism), disruption of cohabitation | 3 (3.53) | 6 (0.80) |
| Other violent behavior + Rioting (other  than exhibitionism), disruption  of cohabitation + Property-related  criminal activity | 0 (0.00) | 2 (0.27) |
| Property-related criminal activity | 12 (14.12) | 82 (10.89) |
| NA | 0 (0.00) | 4 (0.53) |
| Sexual violence or other acts against  children (possession of child pornography, etc.) | 0 (0.00) | 88 (11.69) |
| Sexual violence against adults | 0 (0.00) | 70 (9.30) |
| Criminal activity related to addictive  substances (manufacturing,  possession, distribution, etc.) | 2 (2.35) | 25 (3.32) |
| Murder or attempted murder | 6 (7.06) | 76 (10.09) |
| Rioting (other than exhibitionism),  disruption of cohabitation) | 29 (34.12) | 182 (24.17) |
| Rioting (other than exhibitionism), disruption  of cohabitation + Crimes against  official decisions (obstruction of official  decision, negligent criminal activity) | 1 (1.18) | 0 (0.00) |
| Rioting (other than exhibitionism), disruption  of cohabitation + Property-related  criminal activity | 1 (1.18) | 4 (0.53) |
| Arson | 3 (3.53) | 8 (1.06) |
